# Is resistance to anti-tuberculosis drugs associated with type 2 diabetes mellitus? A register review in Beijing, China

**DOI:** 10.3402/gha.v7.24022

**Published:** 2014-05-19

**Authors:** Fengling Mi, Guanglu Jiang, Jian Du, Liang Li, Wentao Yue, Anthony D. Harries, Sven Gudmund Hinderaker, Yan Lin

**Affiliations:** 1 Department of Research, Beijing Chest Hospital, Capital Medical University/Beijing Tuberculosis and Thoracic Tumor Research Institute, Beijing, China; 2 National Tuberculosis Clinical Laboratory, Beijing Chest Hospital, Capital Medical University/Beijing Tuberculosis and Thoracic Tumor Research Institute, Beijing, China; 3 Administration Office, Clinical Center on Tuberculosis, China CDC, Beijing Chest Hospital, Capital Medical University/Beijing Tuberculosis and Thoracic Tumor Research Institute, Beijing, China; 4 Department of Research, International Union Against Tuberculosis and Lung Disease, Paris, France; 5 Department of Infectious Diseases and Tropical Medicine, London School of Hygiene and Tropical Medicine, London, UK; 6 Center for International Health, University of Bergen, Bergen, Norway; 7 International Union Against Tuberculosis and Lung Disease, Beijing, China

**Keywords:** diabetes mellitus, diabetes duration, diabetes control, tuberculosis, multidrug-resistant tuberculosis, China

## Abstract

**Background:**

China has a high burden of drug-resistant tuberculosis (TB) and diabetes mellitus (DM).

**Objective:**

The objectives of this study were to determine the following in patients with culture-confirmed TB: 1) demographic characteristics and disease patterns in relation to the presence or absence of type 2 diabetes and 2) presence or absence of drug resistance to isoniazid (INH), rifampicin (RMP) or both in relation to duration of diabetes and control of diabetes.

**Design:**

This is a cross-sectional and retrospective study involving record reviews.

**Results:**

There were 621 patients with culture-positive TB, of whom 187 (30%) had previously known or new type 2 diabetes. In those with diabetes, there was a significantly higher proportion of males, persons aged ≥35 years and patients registered with new TB (*p*<0.05). Prevalence of multidrug-resistant TB (MDR-TB) was 6.2% in new patients (*N*=422) and 62.3% in previously treated patients (*N*=199), with no significant differences between those with and without diabetes. In patients with diabetes, there was no association of drug resistance with diabetes duration or disease control [assessed by fasting blood glucose (FBG) at 1 week].

**Conclusion:**

A high proportion of patients with TB in a tertiary health facility, Beijing, China, had diabetes, but there was no association between type 2 diabetes and drug-resistant TB. Further prospective studies are needed to confirm these findings.

Drug-resistant tuberculosis (TB), especially multidrug-resistant TB [MDR-TB, defined as resistance to at least isoniazid (INH) and rifampicin (RMP)], is a major threat to the control of TB worldwide (1). China is one of the high TB and MDR-TB burden countries in the world, with the second largest absolute number of MDR-TB patients (2). Although China has achieved the 2005 global TB target (70% case detection and 85% treatment success), the problem of drug-resistant TB is a serious obstacle to achieving the Millennium Development Goal (MDG) of halving TB prevalence and deaths by 2015 (3, 4).

As a consequence of population growth, ageing, changing lifestyles and urbanisation, China is also witnessing an escalating epidemic of diabetes mellitus (DM) (5, 6). Available data from a recent study in a nationally representative sample of the population show the age-standardised prevalence of DM and prediabetes to be 9.7 and 15.5%, respectively, which extrapolates to 92 million adults with DM and 148 million with prediabetes in the country (7).

DM is a well-known risk factor for TB. It increases the risk of developing active TB by a factor of 2–3 compared with the normal population (8). There are also reports of DM patients with TB being more likely to develop drug-resistant TB (9–11), although the numbers of patients reported in these studies are small, and there are few data on whether the duration of DM and control of disease have any association with the presence of drug resistance.

The double burden of disease is a serious and growing challenge for Chinese health systems, and DM poses a threat for TB control in China. However, there is little information about whether DM, duration of disease and control of DM have any association with drug-resistant TB. The objectives of this study, therefore, were to describe in patients aged 15 years and above with TB registered in Beijing Tuberculosis and Thoracic Tumor Research Institute: 1) demographic characteristics and patterns of TB in relation to the presence or absence of type 2 DM, and 2) the presence or absence of drug resistance to INH, RMP or both in relation to duration of DM and quality of DM control.

## Methods

### Study design

This was a cross-sectional and retrospective study involving a record review of TB patients registered and treated in Beijing Tuberculosis and Thoracic Tumor Research Institute, China.

### Setting – general and study site

#### General

China is a country of 1.3 billion people, with approximately 90 million people living with DM and each year about 1 million estimated new cases of TB and 100,000 new incident cases of MDR-TB (2).

#### Study site

The study site was Beijing Tuberculosis and Thoracic Tumor Research Institute. Beijing is the capital city of China, which lies in the north-east of China, with a population of 19.6 million. Beijing Tuberculosis and Thoracic Tumor Research Institute houses 25 clinical and technical departments and 8 scientific research departments, and there is an integrated system for diagnosis, treatment and basic scientific research on TB and thoracic tumour. The institute had previously coordinated and implemented the National Survey of drug-resistant TB in the country (12), and had participated in a study of bidirectional screening of DM and TB in China (13, 14). There are 955 staff members, a total of 533 inpatient beds of which 210 are available for TB patients. There are about 800 TB patients admitted for inpatient care each year, and of these, 450 have culture-confirmed pulmonary disease. Patients have to pay for diagnosis and treatment in the hospital.

Diagnosis of TB. The diagnosis of TB is made in line with China's TB Control Programme Guidelines (4). Bacteriological tests for the diagnosis of TB include smear, culture and drug susceptibility testing (DST). Each inpatient is investigated by sputum smear and culture at the time of hospitalisation, and if the culture is positive for *Mycobacterium tuberculosis*, then DST is performed. Smears are stained with 0.1% auramine-O and read with LED microscopy (4), and a diagnosis of smear-positive pulmonary TB is normally made within 3 days. For mycobacterial culture, sputa are digested with 4% sodium hydroxide (NaOH) for 15 min and then inoculated to acid-buffer Lowenstein-Jensen (LJ) media (4). Drug resistance testing is done using the proportion method (LJ media) (15), with concentrations of INH and RMP being 0.2 and 40 µg/ml, respectively. The result of an external quality assessment (EQA) of DST by the TB National Reference Laboratory in 2012 showed that the accordance rates for both INH and RMP were above 90%. Once patients are diagnosed with TB, they are categorised as either new or retreatment cases according to the patient's treatment history.

Diagnosis for DM. The diagnosis of type 2 DM follows national guidelines which stipulates that a fasting blood glucose (FBG) test is carried out using venous plasma and a biochemical analyzer with a cut-off threshold in line with that recommended by the World Health Organization (WHO) (16). The diagnosis is based on the following: (1) determining whether patients are already known to have type 2 DM, (2) in those with no known history of DM, measuring an FBG (14), and (3) in those with previously known type 2 DM, measuring an FBG and asking the patient the duration of type 2 DM. As part of routine assessments, every inpatient is asked to undergo FBG. For a diagnosis of type 2 DM to be made, a patient has to have two FBG measurements ≥7.0 mmol/L (126 mg/dL) at different time intervals. The diagnosis of type 2 DM in newly identified patients usually takes 1 week. Newly diagnosed type 2 DM patients are defined as having a duration of disease less than or equal to 1 month while previously known type 2 DM patients are defined as having a duration of disease from the time that type 2 DM was first diagnosed providing that this was longer than 1 month.

### Patients

All inpatients aged 15 years or older and registered with mycobacterial culture-confirmed TB in Beijing Tuberculosis and Thoracic Tumor Research Institute between 1 January 2011 and 30 June 2012 (18 months) were included in the study.

### Data sources, data variables and data collection

Data were obtained from the medical records, the drug-resistant TB register and the electronic system of medical records in Beijing Tuberculosis and Thoracic Tumor Research Institute. Data variables included: 1) TB status: registration number of the medical record; date of registration; age; sex; ethnic nationality group (the Han ethnic group and others); occupation (manual worker, office worker, farmer, unemployed worker and others); residence (a Beijing resident and others); category of TB (new or previously treated); history of TB treatment with one of four drugs (INH; RMP; ethambutol, EMB; and pyrazinamide, PZA); 2) drug-resistant status of TB: no resistance; resistance to INH; resistance to RMP; MDR-TB; 3) DM status at the time of TB registration: diagnosis of type 2 DM (Yes=previously known or new diagnosis; No=none of these) for those with type 2 DM, the measurement of FBG in the first week of treatment and the duration of disease from when it was first diagnosed. Data were collected into a paper proforma and an EXCEL electronic tool by six staff members (four resident physicians, two researchers) of Beijing Tuberculosis and Thoracic Tumor Research Institute.

### Analysis and statistics

Descriptive analysis was performed. Comparisons were made for patterns of TB in patients with and without type 2 DM. Comparisons were made between drug-sensitive and drug-resistant TB in relation to quality of DM control as determined by FBG and duration of DM. DM control was arbitrarily defined as: DM good control, FBG <7.0 mmol/L; DM poor control, FBG 7–10 mmol/L; DM bad control, FBG >10 mmol/L. Statistical comparisons between categorical variables were made using the chi-square test for univariate analysis followed by multivariate regression. In logistic regression models, all variables that were significant in the univariate analysis were adjusted for using odds ratios (OR) and 95% confidence intervals (95% CI). Levels of significance were set at 5%.

## Results

### Demographic characteristics, patterns of disease and drug resistance profiles of TB patients with and without type 2 DM

There were 621 patients with culture-positive TB. With the exception of one patient with no information about DM, there were 187 (30%) patients with type 2 DM. Demographic characteristics, patterns of disease and drug resistance profiles of TB patients with and without type 2 DM are shown in Table 1. On univariate analysis, males, persons aged 35 years and above, manual workers and Beijing residents with TB had a higher prevalence of DM compared with their reference population. Similarly, there was a higher proportion of patients with new TB compared with previously treated TB in those with DM. The results of multivariate analysis are also shown in Table 1, with males, persons aged 35 years and above, Beijing residents and new patients having a significantly higher prevalence of type 2 DM. Prevalence of single drug resistance to INH or RMP and MDR-TB was 10.0 and 6.2%, respectively, in new patients (*N*=422) and 12.0 and 62.3%, respectively, in previously treated patients (*N*=199), with no significant differences between those with and without type 2 DM.

**Table 1:** Characteristics of TB patients with and without type 2 DM, Beijing, China

Characteristics	Total number, *N*=621	TB with DM number (%), *N*=187	Univariate OR (95% CI)	Adjusted OR (95% CI)
Demographic characteristics				
Sex				
Female	171	28 (16.4)	Reference	
Male	450	159 (35.3)	2.8 (1.8–4.4)	2.1 (1.3–3.4)
Age				
15–34	155	11 (7.1)	Reference	
35–54	196	82 (41.8)	9.4 (4.8–18.5)	7.7 (3.8–15.5)
≥55	270	94 (34.8)	7.0 (3.6–13.6)	5.0 (2.5–10.0)
Ethnic group				
Han ethnic group	591	180 (30.5)	Reference	
Others	30	7 (23.3)	0.7 (0.3–1.6)	
Occupation				
Office worker	86	22 (25.6)	Reference	
Manual worker	90	40 (44.4)	2.3 (1.2–4.4)	0.6 (0.3–1.2)
Farmer	110	30 (27.3)	1.1 (0.6–2.1)	0.5 (0.3–1.0)
Unemployed worker	139	39 (28.1)	1.1 (0.6–2.1)	0.7 (0.4–1.3)
Others	193	56 (29.0)	1.2 (0.7–2.1)	0.6 (0.4–1.1)
Resident				
Beijing resident	335	131 (39.1)	Reference	
Others	286	56 (19.6)	0.4 (0.3–0.6)	0.6 (0.4–0.9)
Category of TB				
New	422	144 (34.1)	Reference	
Previously treated	199	43 (21.6)	0.5 (0.4–0.8)	0.6 (0.4–0.9)
Drug resistance				
Patients with new cases of tuberculosis				
Susceptibility to isoniazid/rifampicin	354	118 (33.3)	Reference	
Isoniazid resistance	37	14 (37.8)	1.2 (0.6–2.5)	
Rifampicin resistance	5	2 (40.0)	1.3 (0.2–8.1)	
Multidrug resistant	26	10 (38.5)	1.3 (0.6–2.8)	
Patients with previously treated tuberculosis				
Susceptibility to isoniazid/rifampicin	51	15 (29.4)	Reference	
Isoniazid resistance	13	5 (38.5)	1.5 (0.4–5.3)	
Rifampicin resistance	11	2 (18.2)	0.5 (0.1–2.8)	
Multidrug resistant	124	21 (16.9)	0.5 (0.2–1.1)	

TB: Tuberculosis; DM: diabetes mellitus; CI: confidence intervals.

### Type 2 DM patients with drug-resistant disease in relation to duration and control of DM

Amongst TB patients with type 2 DM, there were 23 patients (12.3%) who were resistant to INH or RMP and 31 patients (16.6%, 31/187) who had MDR-TB. In those with type 2 DM, there was no statistical association between duration of disease and patterns of pan-sensitive, drug-resistant or MDR-TB, when comparing patients with duration of disease for less than 1 month to those with a longer duration (Table 2). Likewise, control of DM as judged by FBG measurements done in the first week of treatment was not statistically different in patients with pan-sensitive, drug-resistant or MDR-TB, when comparing patients with good DM control to those with poor or bad DM control (Table 2).

**Table 2:** Patients with and without drug-resistant TB in relation to duration and control of diabetes, Beijing, China

			Single-drug resistant TB^a^		Multidrug-resistant TB^b^	
			
DM patients	Number of patients	Pan-sensitive TB number (%)	Number (%)	Versus pan-sensitive TB Odds ratio (95% CI)	Number (%)	Versus pan-sensitive TB Odds ratio (95% CI)
DM duration						
DM duration ≤1 month	44	34 (77.2)	5 (11.4)	Reference	5 (11.4)	Reference
DM duration >1 month and <5 years	57	36 (63.1)	9 (15.8)	1.7 (0.5–5.6)	12 (21.1)	2.3 (0.7–7.1)
DM duration ≥5 years	86	63 (73.2)	9 (10.5)	1.0 (0.3–3.1)	14 (16.3)	1.5 (0.5–4.6)
DM quality control						
DM good control^c^	49	33 (67.3)	7 (14.3)	Reference	9 (18.4)	Reference
DM poor control^d^	70	48 (68.6)	11 (15.7)	1.1 (0.4–3.1)	11 (15.7)	0.8 (0.3–2.3)
DM bad control^e^	68	52 (76.5)	5 (7.3)	0.5 (0.1–1.6)	11 (16.2)	0.8 (0.3–2.1)

DM: diabetes mellitus; TB: tuberculosis.

a Single-drug-resistant TB=resistant to either isoniazid or rifampicin.

b Multidrug-resistant TB=resistant to at least isoniazid and rifampicin.

c DM good control: Fasting blood glucose ≤7.0 mmol/L in first week of treatment.

d DM poor control: Fasting blood glucose 7.1–10.0 mmol/L in first week of treatment.

e DM bad control: Fasting blood glucose >10.0 mmol/L in first week of treatment.

## Discussion

This is one of the first studies in Beijing, China, to assess the relationship between type 2 DM and drug-sensitive/drug-resistant TB in patients with culture-confirmed *M. tuberculosis*. TB patients with type 2 DM were more likely to be male, older than 35 years of age, a manual worker, a Beijing resident and to have new TB compared with those who did not have DM. Otherwise, we found no significant association between type 2 DM and the patterns of drug-sensitive/drug-resistant TB, and in those with type 2 DM no association between drug-sensitive/drug-resistant TB and duration of DM or quality of DM control.

The strengths of this study were the large number of TB patients who were consecutively registered and enrolled to treatment in a high-level hospital in China. All patients were investigated in the same manner with smears, cultures of *M. tuberculosis* and DST carried out in a quality-assured laboratory using methods that were in line with WHO guidelines. Tests for FBG were carried out in all patients in line with the routine practice in the hospital. The data were collected through the electronic system and checked with the information recorded in paper-based registers, so we believe that the information is credible. The conduct of this observational study and the study report also followed STROBE guidelines (17).

Limitations related to the operational nature and cross-sectional design of the study. All patients were hospitalised, as it would have been difficult to get all tests done and recorded on outpatients, but this introduces an element of bias into the study population as more patients would have been sick compared with TB patients in general and they also had to pay for diagnosis and treatment. Thus, our patients may not have been representative of the general population as poor patients would not have been included. A further limitation was that DM control was based on measurements of FBG, whereas measurements of glycated haemoglobin (HbA1C) would have been better and would have provided a more reliable assessment of DM control. However, HbA1C is expensive, and although some patients did have this test performed in the hospital setting, the majority did not. Finally, the cross-sectional design of our study limits the conclusions that can be drawn about associations between DM and drug-resistant TB. In contrast with the findings from our study, there was a previous study from China reporting that the frequency of MDR-TB among DM patients with TB was higher than that among TB patients without DM (17.7 versus 8.4%, *p<*0.01) (10). Several other studies have also shown this association (11, 18), with one of these studies even suggesting that patients with DM may be eight times more likely to have infection with MDR-TB (18). In Eastern Taiwan, there was a recent paper reporting an association between DM and INH resistance in new and previously treated TB patients, but no association was found between DM and MDR-TB (19). Reasons for the association of DM and drug resistance are speculative and may be because DM patients do not achieve or maintain adequate blood levels of RMP as a result of drug–drug interactions between anti-TB drugs and oral hypoglycaemic drugs, bacterial genetics, strain mutations and immune impairment as a result of DM (11, 20, 21). It might also be expected that with delayed sputum culture conversion and increased rates of treatment failure (22, 23), which have been documented in cohorts of TB patients with DM, that DM patients with recurrent or previously treated TB might be at a higher risk of drug resistance and MDR-TB.

A systematic review of four studies, however, found no association between DM-TB and drug-resistant TB in patients with recurrent disease (22). In countries such as India, Japan and USA, there have also been no studies documenting a positive association between recurrent drug-resistant TB and MDR-TB when DM patients are compared with non-DM patients (11, 19, 24–26). Indeed, there have been reports of a lower prevalence of drug resistance in TB patients with DM (27).

It is possible that these different and contrasting findings in generally well-conducted studies are because other important determinants of TB treatment outcomes – that include death, failure and recurrent disease – and their linkage to the possibility of drug resistance have not been factored in when comparing DM with no DM. For example, there is an established association between cigarette smoking and TB treatment outcomes including an increased risk of death (28) and an increased risk of relapse after successful completion of treatment (29). A recent study from Korea showed that DM and smoking independently increased the risk of death in TB patients, with the combined impact of both determinants yielding a hazard ratio for death of almost six (30).

While this current study suggests that there is no increased risk of drug-resistant TB in patients with type 2 DM, it would be unwise to be complacent on this issue. There is a sizeable and growing burden of drug-resistant TB in China, and this has implications for the management of DM patients. Good infection control measures must be adopted and practiced in DM clinics to prevent any nosocomial spread of TB, and DM patients who are responding poorly to anti-TB treatment should be considered and investigated for drug-resistant disease. Further prospective research using a cohort design with adequate follow-up is needed, which includes all known determinants for drug-resistant TB in DM patients so that a better understanding is reached about the interactions between the two diseases.

## Conclusion

In Beijing, China, hospitalised TB patients with DM were more likely to be male, older, a Beijing resident, of worker occupation and to have new TB compared with TB patients who did not have type 2 DM. We found no association between type 2 DM and drug-sensitive/drug-resistant TB, and in those with type 2 DM there was no association between drug-resistant status and duration of DM or control of DM, the latter determined by FBG in the first week of anti-TB treatment. Further prospective research is needed on the association between DM and drug-resistant TB, and this research will need to factor in other determinants of drug resistance in order to better understand the interactions between the two diseases.
